# Body axis elongation and ciliary function in zebrafish require Noni a novel ciliary protein

**DOI:** 10.1186/2046-2530-1-S1-P72

**Published:** 2012-11-16

**Authors:** J Jerber, CB Chhin, SF Soulavie, BD Baas, VC Vesque, DB Durand, TJ Thomas

**Affiliations:** 1 University of Lyon, France

## 

Cilia are highly conserved structures found from protozoa to mammals where they play major functions in motility and sensation. Ciliary dysfunction leads to a variety of human genetic diseases called ciliopathies. RFX transcription factors are key players in regulating genes involved in cilia assembly from *C. elegans* to mammals. Using a genomic screen to identify targets of RFX, we selected a novel gene, noni, potentially implicated in cilia biology. No known function has been described for this gene, which is highly conserved and specific of ciliated species. We show that noni expression is increased during in vitro induced ciliogenesis in primary mouse ependymal cell culture and that Noni localizes to cilia from *Drosophila* to mammals. We performed functional analysis in zebrafish and show that loss of function of noni mimics human symptoms associated with ciliopathies such as kidney cysts and randomized left right patterning. We demonstrate that Noni is necessary for cilia function. Indeed, we show that nodal flow is impaired in Kupffer’s vesicle of noni morphants. Moreover, noni morphants have defects in body axis elongation and present impaired basal body positioning in the floor plate suggesting that noni is implicated in planar cell polarity (PCP). To test this hypothesis, we are currently analysing genetic interaction with known PCP components associated with cilia function. In conclusion, we have identified a novel conserved ciliary gene noni, which is necessary for ciliary function and planar cell polarity in zebrafish.

